# Implementation of the national antimicrobial stewardship competencies for UK undergraduate healthcare professional education within undergraduate pharmacy programmes: a survey of UK schools of pharmacy

**DOI:** 10.1093/jacamr/dlad095

**Published:** 2023-08-08

**Authors:** Ryan A Hamilton, Molly Courtenay, Kevin J Frost, Roger Harrison, Helen Root, David G Allison, Antonella P Tonna, Diane Ashiru-Oredope, Mamoon A Aldeyab, Katherine Shemilt, Sandra J Martin

**Affiliations:** School of Pharmacy, De Montfort University, Leicester LE1 9BH, UK; School of Healthcare Sciences, Cardiff University, Cardiff CF24 0AB, Wales; Pharmacy Department, Airedale NHS Foundation Trust, Keighley BD20 6TD, UK; School of Health Sciences, Faculty of Biology, Medicine and Health, The University of Manchester, Manchester M13 9PT, UK; School of Pharmacy, De Montfort University, Leicester LE1 9BH, UK; Division of Pharmacy & Optometry, The University of Manchester, Manchester M13 9PT, UK; School of Pharmacy and Life Sciences, Robert Gordon University, Aberdeen AB10 7AQ, UK; School of Pharmacy, University College London, London WC1N 1AX, UK; UK Health Security Agency, London SW1P 3JR, UK; Department of Pharmacy, University of Huddersfield, Huddersfield HD1 3DH, UK; School of Pharmacy and Biomolecular Sciences, Liverpool John Moores University, Liverpool L3 3AF, UK; School of Pharmacy & Medical Sciences, University of Bradford, Bradford BD7 1DP, UK

## Abstract

**Background:**

Pharmacists play a key role in antimicrobial stewardship (AMS). Consensus-based national AMS competencies for undergraduate healthcare professionals in the UK reflect the increasing emphasis on competency-based healthcare professional education. However, the extent to which these are included within undergraduate pharmacy education programmes in the UK is unknown.

**Objectives:**

To explore which of the AMS competencies are delivered, including when and at which level, within UK undergraduate MPharm programmes.

**Methods:**

A cross-sectional online questionnaire captured the level of study of the MPharm programme in which each competency was taught, the method of delivery and assessment of AMS education, and examples of student feedback.

**Results:**

Ten institutions completed the survey (33% response rate). No institution reported covering all 54 AMS competencies and 5 of these were taught at half or fewer of the institutions. Key gaps were identified around taking samples, communication, outpatient parenteral antimicrobial therapy and surgical prophylaxis. The minimum time dedicated to AMS teaching differed between institutions (range 9–119 h), teaching was generally through didactic methods, and assessment was generally through knowledge recall and objective structured clinical examinations. Feedback from students suggests they find AMS and antimicrobial resistance (AMR) to be complex yet important topics.

**Conclusions:**

UK schools of pharmacy should utilize the competency framework to identify gaps in their AMS, AMR and infection teaching. To prepare newly qualified pharmacists to be effective at delivering AMS and prescribing antimicrobials, schools of pharmacy should utilize more simulated environments and clinical placements for education and assessment of AMS.

## Introduction

Pharmacists play a key role in the multidisciplinary approach to antimicrobial stewardship (AMS). They are responsible for clinical review and optimization of antimicrobial therapies, intervening on suboptimal prescribing, ensuring accurate and safe dispensing and supply to health systems and patients, monitoring prescribing patterns and educating other health professionals, patients and the public, as well as leading local and national AMS programmes.^1–4^

The central role of pharmacists in AMS highlights the need to develop knowledge and competence in these topics through undergraduate pharmacy education. This has been recognized by the WHO, which published a curricula guide for *Health workers’ education and training on antimicrobial resistance* that makes specific reference to pharmacists.^5^ Knowledge and competence of AMR and AMS will become imperative as the General Pharmaceutical Council (GPhC) Standards for the Initial Education and Training Standards (IETS) of Pharmacists in the UK will see student pharmacists become prescribers at the point of registration from Summer 2026 onwards (See Supplementary information S1, available as Supplementary data at *JAC-AMR* Online, for more detail on UK pharmacy undergraduate MPharm programmes).^6^

In 2016, a cross-sectional survey of undergraduate programmes in human and veterinary medicine, dentistry, pharmacy and nursing in the UK was undertaken, which demonstrated that AMS principles were included in most programmes.^7^ However, this varied and the authors recommended standardization of AMS curricula. A survey of US pharmacy schools and colleges (*n* = 116) in 2017 reported that 68.1% of courses included AMS teaching as a required element of the curricula, focusing on definitions, principles and the role of pharmacist.^8^

Consensus-based national AMS competencies for undergraduate healthcare professionals in the UK^9^ were developed that built on the antimicrobial prescribing and stewardship competencies published by PHE (now the UK Health Security Agency).^10^ This AMS competency framework contains six domains (Table 1) with 54 descriptors (see Table 2) that reflect the increasing emphasis on competency-based healthcare professional education, which focuses on the learner’s ability to successfully carry out tasks in the real world, rather than absorbing and reciting content.^11^

**Table 1.** Domains and competency statements for the AMS framework for undergraduate healthcare professionals in the UK (Courtenay *et al.*^9^)

Domain number	Domain title	Competency statement
1	Infection prevention and control	All qualified healthcare professionals must understand the core knowledge underpinning infection prevention and control, and use this knowledge to appropriately prevent the spread of infection
2	Antimicrobials and AMR	All qualified healthcare professionals need to understand the core knowledge underpinning the concept of AMR and use this knowledge to help prevent AMR
3	The diagnosis of infection and the use of antibiotics	All qualified healthcare professionals need to demonstrate knowledge in how infections are diagnosed and the appropriate use of antimicrobials and use this knowledge appropriately to support the accurate diagnosis of infection and the appropriate use of antimicrobials
4	Antimicrobial prescribing practice	All qualified healthcare professionals need to be aware of how antimicrobials are used in practice in terms of their dose, timing, duration and appropriate route of administration, and apply this knowledge as part of their routine practice
5	Person-centred care	All qualified healthcare professionals must seek out, integrate and value as a partner the input and engagement of the patient/carer in designing and implementing care
6	Interprofessional collaborative practice	All qualified healthcare professionals need to understand how different professions collaborate in relation to how they contribute to AMS

**Table 2.** Competency framework and heatmap of schools reporting at which level taught

Domain	Competency statement		Number of schools teaching at each level^a^				
			L4	L5	L6	L7	Not taught
Infection prevention and control	1	Describe what a microorganism is	9	6	1	0	0
	2	Describe the different types of organisms that may cause infections	8	6	5	6	0
	3	Explain what an antimicrobial-resistant organism is	6	7	7	6	0
	4	Explain the ‘chain of infection’	4	4	6	3	0
	5	Define the components required for infection transmission (i.e. presence of an organism, route of transmission of the organism from one person to another, a host who is susceptible to infection)	6	6	6	4	0
	6	Describe the routes of transmission of infectious organisms, i.e. contact, droplet, airborne routes	5	8	5	5	0
	7	Present and recognize the characteristics of a susceptible host	5	7	3	7	1
	8	Demonstrate an understanding of the importance of surveillance	3	4	3	4	0
	9	Describe how vaccines can prevent infections in susceptible persons	5	5	6	5	0
	10	Demonstrate the application of standard precautions in healthcare environments	3	6	4	6	0
	11	Apply appropriate policies/procedures and guidelines when collecting and handling specimens	4	4	1	3	2
	12	Apply policies, procedures and guidelines relevant to infection control when presented with infection control cases and situations	2	4	3	3	4
	13	Implement work practices that reduce risk of infection (such as taking appropriate immunization or not coming to work when sick to ensure patient and other healthcare worker protection)	5	6	6	5	2
	14	Appreciate that healthcare workers have the accountability and obligation to follow infection control protocols as part of their contract of employment	7	5	4	5	3
	15	Act as a role model to healthcare workers and members of the public by adhering to infection prevention and control principles	4	3	3	4	3
	16	Demonstrate knowledge and awareness of international/national strategies on infection prevention and control and AMR such as Global Action Plan for AMR and Save Lives—Clean Your Hands http://www.who.int/gpsc/5may/en/ and the UK Government’s 5-year Antimicrobial Resistance Strategy	2	2	3	6	2
Antimicrobials and AMR	17	Recognize the symptoms of infection	6	7	7	7	0
	18	Describe at least two different ways that antimicrobials may kill bacteria	5	6	4	5	0
	19	Discuss how inappropriate antimicrobial use (including non-adherence to treatment regime) may lead to AMR	5	4	6	8	0
	20	Identify approaches to support optimal prescribing of antimicrobials	2	4	7	7	0
Antimicrobial prescribing and stewardship	21	Explain how microbiology samples may aid diagnosis of infection	5	5	7	6	0
	22	Describe how and demonstrate (following local procedures) the appropriate taking of samples	1	0	1	0	8
	23	Interpret microbiology results/reports from the laboratory at a basic level	4	6	4	7	0
	24	Explain why self-limiting bacterial or viral infections are unlikely to benefit from antimicrobials	5	4	6	5	0
	25	Describe and demonstrate the self-management strategies required to treat self-limiting infections (i.e. analgesia/rest/fluids)	4	4	5	7	0
	26	Understand the importance of following local antimicrobial policies (i.e. their development is based on local resistance patterns) and follow these policies in practice	1	3	8	10	0
	27	Explain the importance of documenting the indications for an antimicrobial (i.e. the route by which it is administered, its duration, dose, dose interval and review date) in clinical notes and demonstrate this in practice	1	2	6	8	0
	28	Demonstrate an understanding of the factors that need to be considered when choosing an antimicrobial (including site of infection and type of bacteria likely to cause an infection at a particular site)	3	4	6	7	0
	29	Describe broad-spectrum and narrow-spectrum antimicrobials and the contribution of broad-spectrum antimicrobials to AMR	2	4	7	7	0
	30	Present and be able to recognize the common side-effects associated with widely administered antimicrobials	2	4	6	8	0
	31	Demonstrate an understanding of why documenting a patient allergy to an antimicrobial is important	1	5	7	9	0
	32	Explain why it is important to consider certain physiological conditions (such as renal function) in patients who receive an antimicrobial	1	4	7	6	0
	33	Describe what is meant by delayed prescribing	0	3	3	4	3
	34	Explain why it is essential that an accurate diagnosis of an allergy to an antimicrobial is based on history and laboratory tests	1	3	4	8	2
Antimicrobial prescribing practice	35	Explain how you would recognize and manage sepsis	1	4	6	6	0
	36	Describe why it is important to use local guidelines to initiate prompt, effective antimicrobial treatment in patients with life-threatening infections	1	3	6	8	0
	37	Describe why it is important to switch from intravenous antimicrobials to oral therapy	1	3	4	8	0
	38	Describe how to switch from intravenous antimicrobials to oral therapy	0	2	3	9	0
	39	Understand the appropriateness of antimicrobial administration models such as outpatient parenteral antimicrobial therapy (OPAT)	1	0	2	3	5
	40	Demonstrate an understanding of the rationale and use of perioperative prophylactic antimicrobials to prevent surgical site infection	1	2	2	3	5
	41	Discuss factors that can influence antimicrobial prescribing and the implications for AMS programmes	2	3	6	5	1
	42	Describe the national guidance on completion of a course of antimicrobials	2	4	4	5	1
	43	Describe some of the medicines with which antimicrobials can sometimes interact	1	5	7	8	0
Person-centred care	44	Support participation of patients/carers, as integral partners when planning/delivering their care	3	5	5	6	3
	45	Share information with patients/carer in a respectful manner and in such a way that is understandable, encourages discussion, and enhances participation in decision-making	5	5	6	8	2
	46	Ensure that appropriate education and support is provided by learners to patients/carer, and others involved with their care or service	3	5	7	7	1
	47	Listen respectfully to the expressed needs of all parties in shaping and delivering care or services	3	6	6	7	2
	48	Discuss patient/carer expectations or demands of antimicrobials and the need to use antimicrobials appropriately	1	3	3	7	3
Interprofessional collaborative practice	49	Demonstrate an understanding of the roles, responsibilities and competencies of other health professionals involved in antimicrobial treatment policy decisions	1	3	3	6	2
	50	Explain why it is important that healthcare professionals involved in the delivery of antimicrobial therapy (including the prescription, delivery and supply) have a common understanding of antimicrobial treatment policy decisions, the quantity of antimicrobial use and effective patient/client outcomes	0	3	2	8	1
	51	Establish collaborative communication principles and actively listen to other professionals and patients/carer involved in the delivery of antimicrobial therapy	2	3	2	4	6
	52	Communicate effectively to ensure common understanding of care decisions	2	4	4	5	4
	53	Develop trusting relationships with patients/carer and other health/social care professionals	3	4	4	6	3
	54	Effectively use information and communication technology to improve interprofessional patient-centred care	1	2	2	5	5

a Relates to Framework for Higher Education Qualifications (FHEQ) level of study on the MPharm, where L4 is first year, L5 is second year, L6 is third year and L7 is the fourth year.

This competency framework has been adapted for nursing courses on an international level^12^ and for UK undergraduate medical students.^13^ Although it is evident that key competencies have been included within UK pre-registration nursing programmes, there are inconsistencies across programmes, and greater knowledge pertaining to the use, management and monitoring of antimicrobials is required.^14^

A recent review found that 86.6% of UK schools of pharmacy included AMS within their curricula^15^ but the extent to which the national AMS competencies for undergraduate healthcare professionals in the UK^9^ are included within undergraduate pharmacy education programmes in the UK is unknown. This research aims to explore the delivery of these AMS competencies within UK undergraduate MPharm programmes, including when, how and at which level within individual programmes.

## Methods

### Ethical consideration

Higher Education Institutes (HEIs) invited to participate in the study (see recruitment, below) were provided with a participant information sheet outlining the purpose, methodology and reporting for the study. Informed consent was obtained on the first page of the online data collection tool (see data collection, below). The University of Bradford provided ethical approval for the study (reference EC26860).

### Design

This was a cross-sectional study using an online questionnaire with 63 questions based upon the consensus-based AMS competencies for UK undergraduate healthcare professional education.^9^

### Recruitment

In 2022, the GPhC listed 33 schools of pharmacy at UK-registered HEIs, all of whom were invited to participate in this study. Initial invitations to participate were sent through the UK Council of University Heads of Pharmacy Schools, who acted as distributors of the initial information about the survey. If no response was received to this initial invitation, then individual heads of schools, programme leads and known subject leads at each school of pharmacy were directly contacted by the research team. A pre-data-collection research meeting was also held to answer initial queries regarding the project and data collection method.

### Data collection

Data were collected between 1 March 2022 and 31 May 2022. To ensure General Data Protection Regulation (GDPR) compliance, the questionnaire was hosted and distributed using the Jisc^™^ online survey platform (see Supplementary information S2) and a unique link for the survey was emailed to each participant. In addition to collecting data about whether the curriculum taught the consensus-based AMS competencies, the questionnaire required participants to indicate (i) the level in the programme in which each competency was taught; (ii) the background of academic staff teaching AMS content; (iii) how this content was delivered and evaluated; (iv) whether or not content would be changed in response to recent global and national AMS initiatives; and (v) examples of student feedback on AMS teaching, learning and assessment. Regular research meetings were hosted by the core research team, which offered the participants support with data collection at their respective institution and addressed any queries. Participants collated data through discussions with colleagues at their HEIs and accessing existing teaching materials prior to completing the online survey.

### Data analysis

Data were checked for completeness and ambiguity before analysis. No data were deemed ambiguous and all data were included. Descriptive statistics were performed on quantitative data through Microsoft Excel (version 2108), to describe trends in delivery of the competencies across institutions and academic years. The minimum time dedicated to teaching AMS at each HEI was calculated as the sum of the lower boundary of the selected time range (as dictated by the survey) per year of study.

Free-text responses relating to student feedback were explored qualitatively by R.A.H., H.R. and S.J.M. to identify key themes. Due to the limited free-text data obtained, it was not appropriate to undertake formal thematic or content analysis, but the principles of thematic analysis^16^ were followed during the identification of key themes. These themes were then refined and finalized by consensus of R.A.H., S.J.M., M.C. and K.J.F.

## Results

Ten institutions (9 in England and 1 in Scotland; 33% response rate) completed the survey and were included in the data analysis. Mean time taken to complete the survey was 21.5 min, range 10–44 min. Nine of these provided data regarding time spent teaching AMS and five provided free-text examples of student feedback (see below).

### AMS competency coverage in the MPharm

No institution reported covering all 54 AMS competencies. The median coverage was 47 competencies (IQR 44.25–49.5). One institution reported covering 53 competencies; the competency *Demonstrate an understanding of the rationale and use of perioperative prophylactic antimicrobials to prevent surgical site infection* (competency 42) was not included in the programme. Conversely, two institutes reported covering 40 of the AMS competencies, which was the minimum number reported.

Twenty-nine (53.7%) of the competencies (Table 2) were taught at every institute. Competencies within the first two domains (*Infection prevention and control* and *Antimicrobials and antimicrobial resistance*) tended to be taught equally across all 4 years of study, whereas the other four domains were predominantly taught in later years of the course.

The least commonly (half or fewer of HEIs) taught competencies were: Competency 22: *Describe how and demonstrate (following local procedures) the appropriate taking of samples* (*n* = 2/10); Competency 51: *Establish collaborative communication principles and actively listen to other professionals and patients/carer involved in the delivery of antimicrobial therapy* (*n* = 4/10); Competency 39: *Understand the appropriateness of antimicrobial administration models such as outpatient parenteral antimicrobial therapy (OPAT)* (*n* = 5/10); Competency 40: *Demonstrate an understanding of the rationale and use of perioperative prophylactic antimicrobials to prevent surgical site infection* (*n* = 5/10); and Competency 54: *Effectively use information and communication technology to improve interprofessional patient-centred care* (*n* = 5/10).

### Pedagogy and delivery

Nine HEIs reported how much time was spent on teaching aligned to the AMS competencies (see Table S1). The mean minimum estimated time spent teaching AMS-related content across all 4 years of the MPharm was 45.7 h (SD ± 33.82 h; range 9–119 h).

Teaching was mostly delivered by generalist pharmacists and non-healthcare professional academics (at 9 of 10 HEIs), followed by antimicrobial pharmacists (*n* = 6), medical practitioners (*n* = 3), nurses (*n* = 1) and academic pharmacists with an interest in AMS (*n* = 1). The inclusion of antimicrobial pharmacists within a teaching team did not impact on the amount of time dedicated to AMS teaching. Case studies were used to teach AMS at all HEIs (*n* = 10), with laboratory practicals (*n* = 9), lectures (*n* = 9) and problem-based learning (*n* = 8) being most common. One HEI reported the use of simulated or virtual environments, while only three HEIs directly involved patients/carers/advocates outside of clinical placements (*n* = 4 in clinical settings).

The most common form of assessment was multiple-choice questions (*n* = 9), followed by short-answer questions (*n* = 8) and objective structured clinical examinations (OSCEs) (*n* = 7). The least commonly used methods of assessments were student presentations (*n* = 3) and placement assessments (*n* = 3).

Five respondents provided free-text responses regarding student feedback on AMS teaching at their institution (Table S2). Of the responses, four were directly related to student feedback and experience, while one was focused on staff/educator perspective. All responses related to pedagogy and two main themes were identified: (i) learning and teaching methods; and (ii) content taught, which was further divided into the subthemes of ‘should be relevant to pharmacist practice’, ‘content is complex’ and ‘AMS teaching is a way of improving awareness of AMR and AMS’ (Table 3).

**Table 3.** Qualitative analysis of free-text responses relating to student feedback on AMS teaching

Theme	Subtheme	Sample quote(s)
Learning and teaching methods	Learning and teaching methods (no subtheme identified)	‘Students enjoyed problem-based learning around complex infections, and professional discussions around practice […] Students preferred the interactive sessions where they developed pharmacist knowledge and implements skills around AMS’ (R10)
Content taught	Should be relevant to pharmacist practice	‘More clinical cases on infection and AMS. More details on IV antibiotics that are prescribed in hospitals. Give us appropriate knowledge about the principle of antibiotic prescribing’ (R4)
	Content is complex	‘Very difficult to teach UG student everything about antimicrobials given the constraints of healthcare courses […] Students sometimes have difficulty understanding the hierarchy of evidence sources in antimicrobial medicine as they are different to other therapeutic areas (patient level better than local better than national etc.)’ (R9)
	AMS teaching is a way of improving awareness of AMR and AMS	‘Give us appropriate knowledge about the principle of antibiotic prescribing. Also helps us to play our role in reducing antibiotic resistance.’ (R4) ‘Overseas students have highlighted how different antimicrobial use is in their country (much increased use) and that it is useful to learn about resistance and how it develops.’ (R8)

[R#] relates to the responder number. See Table S2 for all feedback.

## Discussion

To our knowledge, this is the first survey of UK undergraduate MPharm programmes and the implementation of the national AMS competencies for UK undergraduate healthcare professional education.^9^ The findings demonstrate variation in the total number of competencies covered and highlights the differences in time dedicated to teaching AMS across HEIs. A key strength of this paper is that it reports delivery of these competencies by year of study. It highlights the likely pedagogical approaches to AMS education on the MPharm, and identifies five competencies (22, 39, 40, 51 and 54) that are covered by half or fewer of responding HEIs.

One limitation is the return rate (33%), which could be explained by the size, complexity and detail of the information required in the survey tool. Another limitation of the survey is it does not measure teaching and assessment against the levels of Miller’s Triangle,^17^ which would align this work more with the GPhC IETS 2021. However, the existing AMS competency framework is not cross-mapped to Miller’s Triangle and capturing this would have increased the size and complexity of our survey. Respondents did provide some feedback on their experiences and perceptions of teaching on AMS; however, there were limited data reflecting student feedback. Future work should survey students on their experiences and perceptions.

Student feedback captured by our study does suggest, however, that students find infections, AMR and AMS to be complex. This concurs with earlier work by Hanna *et al.*,^18^ who showed that 61.6% (*n* = 112) of final-year pharmacy students felt confident discussing AMS with patients or other healthcare professionals. These findings also align with work by Inácio *et al*.^19^ and Dyar *et al*.,^20^ who identified that a significant proportion of pharmacy students show a lack of understanding of the fundamentals of AMR mechanisms and AMS policies.

Whilst 29 competencies were covered at every school that responded, five competencies were covered by half or fewer of responding HEIs. Pharmacists play a key role in delivering AMS across healthcare,^3^ which is most effective when successful interprofessional working relationships with other health professionals is established.^21–23^ It is therefore concerning that two of the competencies less frequently covered in the MPharm pertain to this important behaviour (Competency 51: *Establish collaborative communication principles and actively listen to other professionals and patients/carer involved in the delivery of antimicrobial therapy*; and Competency 54: *Effectively use information and communication technology to improve interprofessional patient-centred care*). While interprofessional working is key for all conditions and clinical settings, it is imperative that pharmacists must be able to work closely with other healthcare professions to educate, support and influence them to uphold the principles of AMS and tackle AMR.^1,3^ Schools of pharmacy can embed these skills through Interprofessional Education (IPE) sessions throughout the MPharm, giving pharmacy students the opportunity to learn with students from other healthcare disciplines, particularly medicine, nursing and dentistry, while focusing on a case relating to the management of infection. This could be delivered and assessed within simulated and clinical environments, enabling demonstration of a range of AMS competencies (e.g. 10, 12, 20, 38—see Table 2) and provide exposure to registered nurses, physicians and surgeons, while supporting attainment of GPhC IETS 2021 Learning outcome 46: *Make use of the skills and knowledge of other members of the multi-disciplinary team to manage resources and priorities.*^6^

The GPhC IETS 2021 set new learning outcomes that have been developed to support pharmacists registering as independent prescribers from Summer 2026 onwards. Pharmacists already prescribe antimicrobials within generalist and specialist services across primary and secondary care,^24^ therefore teaching will need to evolve to incorporate prescribing skills, including decision-making, relating to antimicrobial therapies and AMS. There is increased emphasis in the GPhC IETS 2021 on clinical and diagnostic skills, including obtaining and interpreting clinical tests. For example, GPhC IETS 2021 Learning outcome 28 states pharmacists can *Demonstrate effective diagnostic skills, including physical examination, to decide the most appropriate course of action for the person*. Only 2 of the 10 HEIs reported teaching students how to take microbiological samples (Competency 22). Embedding this AMS competency is likely to bear greater clinical importance as point-of-care testing becomes more commonplace within community pharmacy settings^25–27^ and alongside the expanding role of pharmacists across primary and secondary care.^28,29^ Therefore, we recommend MPharm programmes embed teaching and assessment (in simulated and clinical environments) regarding key microbiological sampling techniques and interpretation, such as swabs of the throat, nasopharynx and wounds, and urine dipstick testing.

The results from this survey also suggest that MPharm programmes may overlook the importance of OPAT (Competency 39) and surgical prophylaxis (Competency 40), and the role of the pharmacist within these. OPAT services have increased in number and size over the past half-decade^30^ and is one of the options to consider when reviewing antimicrobial therapy within secondary care,^31^ whereas surgical antimicrobial prophylaxis has been an incentivized focus of quality improvement within the NHS,^32^ demonstrating the importance of these competencies for pharmacists and pharmacy students. Aside from AMS, pharmacy student education relating to OPAT can be underpinned with the pharmaceutical principles of drug stability and storage, optimizing pharmacokinetic profiles, and patient monitoring, lending itself to problem-based learning (PBL) and even laboratory pedagogy (discussed below).

It should be borne in mind that HEIs may be teaching AMS topics that are not included within the competency framework, therefore may not have been captured within the survey. Future work should review this competency framework and consider adaptations required for pharmacists, similar to what was undertaken for nursing and medical education.^12,13^ An adapted competency framework should be mapped to the outcomes set by the GPhC IETS 2021 and indicate level of competence to be assessed in line with Miller’s Triangle.^17^

There was wide variation in the time spent between HEIs in teaching AMS. While our study cannot derive the impact of this on AMS knowledge, attitudes and competence of pharmacy graduates, our study suggests that AMS may not receive the attention it deserves at some schools of pharmacy. Seaton *et al.*^33^ reported high capability and awareness, positive attitudes and strong motivation regarding AMR and AMS amongst academic pharmacists (*n* = 4); thus, a lack of understanding regarding the importance of AMS and AMR is not likely to be driving the differences in time spent delivering AMS education observed in our survey.

Results from our survey (Table 2) reinforce the spiral curriculum concept for undergraduate pharmacy education in the UK.^6^ Students learn the underpinning science knowledge in early years,^34^ which is then applied to case-based scenarios and clinical practice in later years. Didactic teaching methods and simple knowledge-recall methods of assessment were commonly employed across HEIs, which was also reported in international research of AMS teaching on pharmacy undergraduate programmes.^8,15^ While most pharmacy schools in our survey employed PBL and OSCE assessments, few taught and assessed in simulated and clinical environments. Pedagogical research demonstrates that participative learning strategies that stimulate engagement reinforce concept understanding and promote retention of knowledge.^34^ For example, PBL broadens and deepens knowledge, increases competence, improves problem-solving and critical evaluation skills, and strengthens interprofessional skills,^35^ all of which can lead to optimal antimicrobial prescribing and improved patient outcomes. The GPhC IETS 2021 places greater emphasis on simulated and in-practice teaching and assessment in order to demonstrate competence, and it will be increasingly important for AMS teaching to be delivered this way also, particularly as the treatment of infections becomes increasingly interprofessional, person-centred and requiring patient education. Moving forward, education and placement providers will need to collaborate on the pedagogical and assessment approaches to ensure MPharm graduates demonstrate the AMS competencies.

It should be noted that this survey was undertaken between 1 March 2022 and 31 May 2022, which is before many MPharm programmes formally adopted the GPhC IETS 2021. Therefore, the results from this survey can be considered a baseline that HEIs can use to review their AMS teaching and utilize the recommendations made above to review their teaching in line with both the AMS competencies and the GPhC IETS outcomes. A similar survey should be undertaken in 5 years’ time, to measure any changes in AMS teaching and assessment since adoption of the GPhC IETS 2021, and consider measuring the level of attainment against Miller’s Triangle.

### Conclusions

All schools of pharmacy should utilize the consensus-based national AMS competencies for undergraduate healthcare professionals in the UK to identify gaps in their teaching, particularly in relation to taking microbiological samples, communication, OPAT and surgical prophylaxis. However, we recommend adaptation of this competency framework to better align with pharmacist education and practice, cross-mapped to the GPhC IETS and Millers’ Triangle. To prepare newly qualified pharmacists to be effective at delivering AMS, schools of pharmacy need to utilize simulated environments and clinical placements for education and assessment of AMS competencies. Future research will be needed to re-evaluate delivery of AMS competencies in MPharm programmes, once the GPhC IETS are fully embedded, capture students’ perceptions of AMS teaching and assessment, and even compare education delivery and outcomes between different health professions.

## Supplementary Material

dlad095_Supplementary_Data
