# Supplementary material for: Implementation of the national antimicrobial stewardship competencies for UK undergraduate healthcare professional education within undergraduate pharmacy programmes: a survey of UK schools of pharmacy
Source: JAC Antimicrob Resist. 2023 Aug 8;5(4):dlad095. doi: 10.1093/jacamr/dlad095 (PMC10409409; doi:10.1093/jacamr/dlad095)
Supplement: dlad095_Supplementary_Data [file dlad095_supplementary_data.docx]

**Supplementary Material**

**Supplementary Information S1: Overview of MPharm in the UK**

In the United Kingdom, the undergraduate pharmacy programme is a four-year undergraduate degree, with an additional 52 weeks of preregistration training and successful completion of an exam prior to registration as a pharmacist. The undergraduate pharmacy degree starts at level 4 in the framework for higher education qualification (FHEQ) and progresses through to level 7 for the degree exit award.

The topics that should be covered within the curriculum of the undergraduate pharmacy programme are outlined in the “Future Pharmacists: Standards for Initial training of pharmacists” (General Pharmaceutical Council, 2011). Suitable topics to help achieve these standards are identified below.

| Topics appropriate to AMS, adapted from the General Pharmaceutical Council (GPhC) “Future Pharmacists: Standards for initial education and training of pharmacists” (2011). | |
| --- | --- |
| Standard | Sub-categories |
| A1.1 How medicines work: | Therapeutics: Infection Control  Applied Physical, Chemical and Biological Sciences: Cell and molecular biology, Microbiology, Immunology |
| A1.2 How people work: | Normal and abnormal structure and function: Infectious diseases and infective processes  Health psychology: disease prevention, health promotion  Objective diagnosis: Differential diagnosis, Objective tests |

In the role as professional regulatory body, the GPhC provides regulations for the initial education and training pf pharmacists and accredits higher education institutes to deliver the training. Each individual school of pharmacy decides how best to deliver the AMS teaching, learning and assessment for their individual undergraduate pharmacy programme. The undergraduate pharmacy curriculum is comprehensive and finding time for AMS in this busy schedule is challenging for academics (is there any type of reference that could support this comment?).

Once qualified, further guidance for prescribers is provided by the antimicrobial prescribing and stewardship post-registration competencies that were developed by Public Health England (PHE, now the UK Health Security Agency) and the Department of Health Expert Advisory Committee on Antimicrobial Resistance and Healthcare Associated Infection (ARHAI) in 2013. These prescribing competencies also complement the Royal Pharmaceutical Society (RPS) generic competency framework for all prescribers and aim to increase the quality of antimicrobial prescribing, which in turn will improve patient care and reduce the development of AMR. As of summer 2026 all newly qualified pharmacists will be independent prescribers. For the legacy workforce, an additional 6 months postgraduate practice-certificate in independent prescribing is required.

**Supplementary Information S2: Data collection tool**

**Antimicrobial stewardship in undergraduate pharmacist education: A national questionnaire survey**

**Page 1: Antimicrobial Stewardship**

Thank you for taking the time to access the survey. Antimicrobial Stewardship (AMS) is identified by the Royal Pharmaceutical Society [1] as an area where pharmacist expertise is important to ensure appropriate use of antibiotics and improve stewardship, in order to reduce antimicrobial resistance.

Improving infection prevention and control practices, optimising prescribing practices and improving professional education are three of the seven aims in the UK National AMR strategy.[2] At the completion of the MPharm degree, students must understand AMS in order to effectively meet the “Apply the principles of clinical therapeutics, pharmacology and genomics to make effective use of medicines for people, including in their prescribing practice” and “Effectively make use of local and national health and social care policies to improve health outcomes and public health, and to address health inequalities” statements within the GPhC education standards [3]

A multi-professional AMS competency framework for undergraduate healthcare professionals [4] was developed in 2018 which has been endorsed by the National Institute for Health and Care Excellence (NICE). This framework comprises 6 domains each of which consist of a competency statement and 5-18 competency descriptors.

We are keen to identify which of these domains/competency descriptors you include in your current undergraduate pharmacist education programme; and who teaches it/how teaching is evaluated. The survey should take you no longer than 15-20 minutes to complete; although you may need to consult with your colleagues prior to completion regarding some of the questions.

By completing this review the results could be used towards curriculum review for your MPharm programme or personal revalidation/CPD

1. The pharmacy contribution to antimicrobial stewardship SEPTEMBER 2017 <https://www.rpharms.com/Portals/0/RPS%20document%20library/Open%20access/Policy/AMS%20policy.pdf> accessed 16 Sep 2021
2. Tackling antimicrobial resistance 2019–2024 Department of Health and Social Care 2019 <https://www.gov.uk/government/publications/uk-5-year-action-plan-for-antimicrobial-resistance-2019-to-2024> accessed 16 Sep 2021
3. Standards for the initial education and training of pharmacists January 2021 <https://www.pharmacyregulation.org/sites/default/files/document/standards-for-the-initial-education-and-training-of-pharmacists-january-2021_0.pdf> accessed 16 Sep 2021
4. Courtenay M, Lim R, Castro-Sanchez E, et al. Development of consensus-based national antimicrobial stewardship competencies for UK undergraduate healthcare professional education. J Hosp Infect.2018 Nov;100(3):245-256. doi: 10.1016/j.jhin.2018.06.022. Epub 2018 Jun 30. <https://doi.org/10.1016/j.jhin.2018.06.022> accessed 16 Sep 2021

**Completing the survey: competencies and descriptors may be taught in several years across the MPharm programme, please tick all that apply.**

**AMS Framework domain 1: Infection Prevention and Control**

Competency statement:

*All qualified healthcare professionals must understand the core knowledge underpinning infection prevention and control and use this knowledge appropriately to prevent the spread of infection.*

**Please indicate which of the following descriptors are included within your undergraduate programme.**

*Descriptors: To support AMS learners must demonstrate infection prevention and control by:*

|  | Not taught | Taught in MPharm Year 1/ FHEQ Level 4 | Taught in MPharm Year 2/ FHEQ Level 5 | Taught in MPharm Year 3/ FHEQ Level 6 | Taught in MPharm Year 4/ FHEQ Level 7 |
| --- | --- | --- | --- | --- | --- |
| 1. Describing what a micro-organism is. |  |  |  |  |  |
| 1. Describing the different types of organisms that may cause infections. |  |  |  |  |  |
| 1. Explaining what an antimicrobial resistant organism is. |  |  |  |  |  |
| 1. Explaining the ‘chain of infection’. |  |  |  |  |  |
| 1. Defining the components required for infection transmission (i.e. presence of an organism, route of transmission of the organism from one person to another, a host who is susceptible to infection). |  |  |  |  |  |
| 1. Describing the routes of transmission of infectious organisms, i.e. contact, droplet, airborne routes. |  |  |  |  |  |
| 1. Present and recognize the characteristics of a susceptible host. |  |  |  |  |  |
| 1. Demonstrate an understanding of the importance of surveillance. |  |  |  |  |  |
| 1. Describe how vaccines can prevent infections in susceptible persons. |  |  |  |  |  |
| 1. Demonstrate the application of standard precautions in healthcare environments. |  |  |  |  |  |
| 1. Apply appropriate policies/procedures and guidelines when collecting and handling specimens. |  |  |  |  |  |
| 1. Apply policies, procedures and guidelines relevant to infection control when presented with infection control cases and situations. |  |  |  |  |  |
| 1. Implement work practices that reduce risk of infection (such as taking appropriate immunization or not coming to work when sick to ensure patient and other healthcare worker protection). |  |  |  |  |  |
| 1. Appreciate that healthcare workers have the accountability and obligation to follow infection control protocols as part of their contract of employment. |  |  |  |  |  |
| 1. Act as a role model to healthcare workers and members of the public by adhering to infection prevention and control principles. |  |  |  |  |  |
| 1. Demonstrating knowledge and awareness of international/national strategies on infection prevention and control and antimicrobial resistance such as Global Action Plan for AMR and Save Lives – Clean Your Hands <http://www.who.int/gpsc/5may/en/> and the UK Government's 5-year Antimicrobial Resistance Strategy. |  |  |  |  |  |

**AMS Framework domain 2: Antimicrobials and antimicrobial resistance**

Competency statement:

*All qualified healthcare professionals need to understand the core knowledge underpinning the concept of antimicrobial resistance and use this knowledge to help prevent antimicrobial resistance.*

**Please indicate which of the following descriptors are included within your undergraduate programme.**

*To support antimicrobial stewardship learners must be able to:*

| (Multiple choice for each row) | Not taught | Taught in MPharm Year 1/ FHEQ Level 4 | Taught in MPharm Year 2/ FHEQ Level 5 | Taught in MPharm Year 3/ FHEQ Level 6 | Taught in MPharm Year 4/ FHEQ Level 7 |
| --- | --- | --- | --- | --- | --- |
| 1. Recognise the symptoms of infection |  |  |  |  |  |
| 1. Describe at least two different ways that antimicrobials may kill bacteria. |  |  |  |  |  |
| 1. Discuss how inappropriate antimicrobial use (including non-adherence to treatment regime) may lead to antimicrobial resistance. |  |  |  |  |  |
| 1. Identify approaches to support optimal prescribing of antimicrobials. |  |  |  |  |  |

**AMS Framework domain 3: The diagnosis of infection and the use of antibiotics**

Competency statement:

*All qualified healthcare professionals need to demonstrate knowledge in how infections are diagnosed and the appropriate use of antimicrobials, and use this knowledge appropriately to support the accurate diagnosis of infection and the appropriate use of antimicrobials.*

**Please indicate which of the following descriptors are included within your undergraduate programme.**

Descriptors: *To support antimicrobial stewardship, learners must be able to:*

| (Multiple choice for each row) | Not taught | Taught in MPharm Year 1/ FHEQ Level 4 | Taught in MPharm Year 2/ FHEQ Level 5 | Taught in MPharm Year 3/ FHEQ Level 6 | Taught in MPharm Year 4/ FHEQ Level 7 |
| --- | --- | --- | --- | --- | --- |
| 1. Explain how microbiology samples may aid diagnosis of infection. |  |  |  |  |  |
| 1. Describe how and demonstrate (following local procedures) the appropriate taking of samples. |  |  |  |  |  |
| 1. Interpret microbiology results/reports from the laboratory at a basic level. |  |  |  |  |  |
| 1. Explain why self-limiting bacterial or viral infections are unlikely to benefit from antimicrobials. |  |  |  |  |  |
| 1. Describe and demonstrate the self-management strategies required to treat self-limiting infections (i.e. analgesia/rest/fluids). |  |  |  |  |  |
| 1. Understand the importance of following local antimicrobial policies (i.e. their development is based on local resistance patterns) and follow these policies in practice. |  |  |  |  |  |
| 1. Explain the importance of documenting the indications for an antimicrobial (i.e. the route by which it is administered, its duration, dose, dose interval, and review date), in clinical notes and demonstrate this in practice. |  |  |  |  |  |
| 1. Demonstrate an understanding of the factors that need to be considered when choosing an antimicrobial (including site of infection and type of bacteria likely to cause an infection at a particular site). |  |  |  |  |  |
| 1. Describe broad-spectrum and narrow-spectrum antimicrobials and the contribution of broad-spectrum antimicrobials to antimicrobial resistance. |  |  |  |  |  |
| 1. Present and be able to recognize the common side-effects associated with widely administered antimicrobials. |  |  |  |  |  |
| 1. Demonstrate an understanding of why documenting a patient allergy to an antimicrobial is important. |  |  |  |  |  |
| 1. Explain why it is important to consider certain physiological conditions (such as renal function) in patients who receive an antimicrobial. |  |  |  |  |  |
| 1. Describe what is meant by delayed prescribing. |  |  |  |  |  |
| 1. Explain why it is essential that an accurate diagnosis of an allergy to an antimicrobial is based on history and laboratory tests. |  |  |  |  |  |

**AMS Framework domain 4: Antimicrobial prescribing practice**

Competency statement:

*All qualified healthcare professionals need to be aware of how antimicrobials are used in practice in terms of their dose, timing, duration and appropriate route of administration, and apply this knowledge as part of their routine practice as follows:*

**Please indicate which of the following descriptors are included within your undergraduate programme.**

Descriptors: *To support antimicrobial stewardship, learners must be able to:*

| (Multiple choice for each row) | Not taught | Taught in MPharm Year 1/ FHEQ Level 4 | Taught in MPharm Year 2/ FHEQ Level 5 | Taught in MPharm Year 3/ FHEQ Level 6 | Taught in MPharm Year 4/ FHEQ Level 7 |
| --- | --- | --- | --- | --- | --- |
| 1. Explain how you would recognize and manage sepsis. |  |  |  |  |  |
| 1. Describe why it is important to use local guidelines to initiate prompt, effective antimicrobial treatment in patients with life-threatening infections. |  |  |  |  |  |
| 1. Describe why it is important to switch from intravenous antimicrobials to oral therapy. |  |  |  |  |  |
| 1. Describe how to switch from intravenous antimicrobials to oral therapy. |  |  |  |  |  |
| 1. Understand the appropriateness of antimicrobial administration models such as outpatient parenteral antimicrobial therapy (OPAT). |  |  |  |  |  |
| 1. Demonstrate an understanding of the rationale and use of perioperative prophylactic antimicrobials to prevent surgical site infection. |  |  |  |  |  |
| 1. Discuss factors that can influence antimicrobial prescribing and the implications for antimicrobial stewardship programmes. |  |  |  |  |  |
| 1. Describe the national guidance on completion of a course of antimicrobials. |  |  |  |  |  |
| 1. Describe some of the medicines with which antimicrobials can sometimes interact. |  |  |  |  |  |

**AMS Framework domain 5: Person-centred care**

Competency statement:

*All qualified healthcare professionals need to be aware of how antimicrobials are used in practice in terms of their dose, timing, duration and appropriate route of administration, and apply this knowledge as part of their routine practice as follows:*

**Please indicate which of the following descriptors are included within your undergraduate programme.**
Descriptors: *To support antimicrobial stewardship, learners must be able to:*

| (Multiple choice for each row) | Not taught | Taught in MPharm Year 1/ FHEQ Level 4 | Taught in MPharm Year 2/ FHEQ Level 5 | Taught in MPharm Year 3/ FHEQ Level 6 | Taught in MPharm Year 4/ FHEQ Level 7 |
| --- | --- | --- | --- | --- | --- |
| 1. 1. Support participation of patients/carers, as integral partners when planning/delivering their care. |  |  |  |  |  |
| 1. 2. Share information with patients/carer in a respectful manner and in such a way that is understandable, encourages discussion, and enhances participation in decision-making. |  |  |  |  |  |
| 1. 3. Ensure that appropriate education and support is provided by learners to patients/carer, and others involved with their care or service. |  |  |  |  |  |
| 1. 4. Listen respectfully to the expressed needs of all parties in shaping and delivering care or services. |  |  |  |  |  |
| 1. 5. Discuss patient/carer expectations or demands of antimicrobials and the need to use antimicrobials appropriately. |  |  |  |  |  |

**AMS Framework domain Six: Interprofessional collaborative practice**

Competency statement:

*All qualified healthcare professionals need to understand how different professions collaborate in relation to how they contribute to antimicrobial stewardship.*

**Please indicate which of the following descriptors are included within your undergraduate programme.**

Descriptors: *To support antimicrobial stewardship, learners must be able to:*

| (Multiple choice for each row) | Not taught | Taught in MPharm Year 1/ FHEQ Level 4 | Taught in MPharm Year 2/ FHEQ Level 5 | Taught in MPharm Year 3/ FHEQ Level 6 | Taught  in MPharm Year 4/ FHEQ Level 7 |
| --- | --- | --- | --- | --- | --- |
| 1. Demonstrate an understanding of the roles, responsibilities, and competencies of other health professionals involved in antimicrobial treatment policy decisions. |  |  |  |  |  |
| 1. Explain why it is important that healthcare professionals, involved in the delivery of antimicrobial therapy (including the prescription, delivery and supply), have a common understanding of antimicrobial treatment policy decisions, the quantity of antimicrobial use, and effective patient/client outcomes. |  |  |  |  |  |
| 1. Establish collaborative communication principles and actively listen to other professionals and patients/carer involved in the delivery of antimicrobial therapy. |  |  |  |  |  |
| 1. Communicate effectively to ensure common understanding of care decisions. |  |  |  |  |  |
| 1. Develop trusting relationships with patients/carer and other health/social care professionals. |  |  |  |  |  |
| 1. Effectively use information and communication technology to improve interprofessional patient-centred care. |  |  |  |  |  |

**BACKGROUND OF ACADEMIC STAFF DELIVERING AMS CONTENT**

What is the background of lecturers delivering AMS (Please tick all that apply):

| Pharmacist (Generalist) |  |
| --- | --- |
| Pharmacist (Antimicrobial Specialist) |  |
| Medical practitioner (e.g. Physician/Surgeon) |  |
| Nurse |  |
| Non-healthcare professional academic (e.g. scientist, pharmacologist, pharmaceutical chemist, non-medical microbiologist) |  |
| Other background |  |

| If other background, please state |  |
| --- | --- |

**DELIVERY OF CONTENT**

**What is the usual (i.e. pre-pandemic) main mode of AMS content delivery (tick all that apply) :**

| Majority Face-to-face taught |  |
| --- | --- |
| Majority On-line learning session |  |
| Blended learning (classroom and on-line activities) |  |
| Other |  |

| If other, please state |  |
| --- | --- |

**What strategies are usually used to deliver AMS content (tick all that apply)?**

| Lectures |  |
| --- | --- |
| Case studies |  |
| Student presentations |  |
| Activities in clinical settings |  |
| Problem based learning |  |
| Use of simulators or other virtual environments |  |
| Laboratory practical sessions |  |
| Discussion with patients/cares/advocates |  |
| Interprofessional education sessions |  |
| E-learning |  |
| Quizzes |  |
| Other |  |

| If other, please state what |  |
| --- | --- |

Your answer should be no more than 200 characters long

**What is the estimated number of hours dedicated to AMS content across the MPharm programme across its four years?**

|  | FHEQ Level 4 / Year 1 | FHEQ Level 5 / Year 2 | FHEQ Level 6 / Year 3 | FHEQ Level 7 / Year 4 |
| --- | --- | --- | --- | --- |
| 1-5 hours |  |  |  |  |
| 6-10 hours |  |  |  |  |
| 11-15 hours |  |  |  |  |
| 16-20 hours |  |  |  |  |
| 21-25 hours |  |  |  |  |
| 26-30 hours |  |  |  |  |
| Over 30 hours |  |  |  |  |

**EVALUATION OF KNOWLEDGE**

**What method(s) of evaluation / assessment are used?**

|  | Yes/No | If yes, are they summative or formative evaluations or a mixture? |
| --- | --- | --- |
| Written coursework (e.g. essays) |  |  |
| Objective structured clinical examination (OSCE) stations |  |  |
| Student presentations |  |  |
| Student portfolio (e.g. reflective practice) |  |  |
| Placement assessment |  |  |
| Short-answer examination |  |  |
| Long-answer examination |  |  |
| Multiple choice question examination |  |  |
| Other |  |  |

**RECENT CHANGES IN TEACHING**

Please choose one selection for the answer to each question

| Do you plan to increase the AMS knowledge taught in your programme in response to the proposed new GPhC initial education standards from 2022? | Yes/No plans made /Not aware of these |
| --- | --- |
| Do you plan to increase the AMS knowledge taught in your programme in response to the UK 5-year action plan for antimicrobial resistance 2019 to 2024 | Yes/No plans made /Not aware of these |
| Do you plan to increase the AMS knowledge taught in your programme in response to the UG Healthcare AMS competencies? | Yes/No plans made/Not aware of these |
| Do you think AMS is currently given enough priority within the MPharm curriculum within your institution? | Yes/No |
| Do you plan to include discussion about the One Health agenda?  *The One Health concept recognises that human health is tightly connected to the health of animals and the environment, for example that animal feed, human food, animal and human health, and environmental contamination are closely linked. Therefore the study of infectious agents that may cross species and environmental barriers to move between these compartments is imperative.* <https://onehealthejp.eu/about> | Yes/No plans made/Not aware of this |

**STUDENT FEEDBACK**

| Have you received any student feedback on AMS teaching? | Yes/No |
| --- | --- |
| If yes, list up to three key messages from this feedback | Free text  Your answer should be no more than 300 characters long. |

Thank you for your participation

**Supplementary Table S1: Estimated minimum number of hours dedicated to teaching AMS content across the MPharm for each individual HEI**

|  | **Estimated number of hours at each level of study at each HEI*** | | | | | | | | |
| --- | --- | --- | --- | --- | --- | --- | --- | --- | --- |
| **HEI No.** | **1** | **2** | **3** | **4** | **5** | **7** | **8** | **9** | **10** |
| Level 4** | 1 – 5 | 21 – 25 | >31 | 16 – 20 | 1 – 5 | 1 – 5 | >31 | 1 – 5 | 11 – 15 |
| Level 5 | 6 – 10 | 11 – 15 | >31 | 16 – 20 | 6 – 10 | 11 – 15 | 6 – 10 | 6 – 10 | >31 |
| Level 6 | 1 – 5 | 6 – 10 | 26 – 30 | 1 – 5 | 1 – 5 | 6 – 10 | 1 – 5 | 11 – 15 | 6 – 10 |
| Level 7 | 11 – 15 | 1 – 5 | >31 | 6 – 10 | 1 – 5 | 11 – 15 | >31 | 6 – 10 | 16 – 20 |
| **Total estimated *minimum**** number of hours across the MPharm** | **19** | **39** | **119** | **39** | **9** | **29** | **69** | **24** | **64** |
| * HEI 6 did not respond to this question  ** Relates to FHEQ level of study on the MPharm where L4 is first year, L5 is second year, L6 is third year, and L7 is the fourth year.  *** Calculated from the number of hours for the lowest boundary of the time range selected by each respondent | | | | | | | | | |

**Supplementary Table S2: Free-text responses regarding feedback from students about AMS teaching**

| **HEI (Responder) Number** | **Have you received any student feedback on AMS teaching?** | **If yes, please list up to three key messages from this feedback:** |
| --- | --- | --- |
| 1 | Yes | 1. Very relevant to practice and the pharmacist’s role in trying to reduce resistance development. 2. Informative and worrying how ineffective antibiotics could become if society does not change the way they are used /prescribed. 3. Puts antibiotic usage into perspective and that everyone has a role to play in combating resistance. |
| 2 | No | n/a |
| 3 | No | n/a |
| 4 | Yes | More clinical cases on Infection and AMS More details on IV antibiotics that are prescribed in hospitals Give us appropriate knowledge about the principle of antibiotic prescribing. Also helps us to play our role in reducing antibiotic resistance |
| 5 | No | n/a |
| 6 | Yes | No response given |
| 7 | No | n/a |
| 8 | Yes | Overseas students have highlighted how different antimicrobial use is in their country (much increased use) and that it is useful to learn about resistance and how it develops. |
| 9 | Yes | Very difficult to teach UG student everything about antimicrobials given the constraints of healthcare courses. Students sometimes have difficulty understanding the hierarchy of evidence sources in antimicrobial medicine as they are different to other therapeutic areas (patient level better than local better than national etc.) |
| 10 | Yes | Students enjoyed problem based learning around complex infections, and professional discussions around practice.; 2nd year students like the practical classes, where the results are then linked to clinical applications.; Students preferred the interactive sessions where they developed pharmacist knowledge and implements skills around AMS. |
